# Memantine administration prevented chorea movement in Huntington’s disease: a case report

**DOI:** 10.1186/s13256-023-04161-z

**Published:** 2023-10-16

**Authors:** Kazumasa Saigoh, Makito Hirano, Yoshiyuki Mitsui, Itsuki Oda, Atsuko Ikegawa, Makoto Samukawa, Keisuke Yoshikawa, Yuko Yamagishi, Susumu Kusunoki, Yoshitaka Nagai

**Affiliations:** 1 Department of Neurology, Faculty of Medicine, Kindai University, Osakasayama, Japan; 2 Department of Life Science, Faculty of Science, and Engineering, Kindai University, 377-2, Ohno-Higashi, Osakasayama, Osaka 589-8511 Japan; 3 Department of Clinical Genetics, Kindai University Hospital, Osakasayama, Japan

**Keywords:** Huntington’s disease, SPECT, Alzheimer’s disease, Memantine, Case report

## Abstract

**Background:**

Huntington’s disease is an autosomal dominant inherited disorder characterized by personality changes (such as irritability and restlessness) and psychotic symptoms (such as hallucinations and delusions). When the personality changes become noticeable, involuntary movements (chorea) also develop. The disease is caused by the CAG repeat expansion in the coding region of the *HTT* gene, and the diagnosis is based on the presence of this expansion. However, there is currently no effective treatment for the progression of Huntington’s disease and its involuntary motor symptoms. Herein, we present a case in which memantine was effective in treating the chorea movements of Huntington’s disease.

**Case presentation:**

A 75-year-old Japanese woman presented to the hospital with involuntary movements of Huntington’s disease that began when she was 73 years old. In a cerebral blood flow test (*N*-isopropyl-*p*-iodoamphetamine–single-photon emission computed tomography), decreased blood flow was observed in the precuneus (anterior wedge) and posterior cingulate gyrus. Usually, such areas of decreased blood flow are observed in patients with Alzheimer’s-type dementia. So, we administered memantine for Alzheimer’s-type dementia, and this treatment suppressed the involuntary movements of Huntington’s disease, and the symptoms progressed slowly for 7 years after the onset of senility. In contrast, her brother died of complications of pneumonia during the course of Huntington’s disease.

**Conclusions:**

We recorded changes in parameters such as the results of the *N*-isopropyl-*p*-iodoamphetamine–single-photon emission computed tomography and gait videos over 7 years. Treatment with memantine prevented the chorea movement and the progression of Huntington’s disease. We believe this record will provide clinicians with valuable information in diagnosing and treating Huntington’s disease.

**Supplementary Information:**

The online version contains supplementary material available at 10.1186/s13256-023-04161-z.

## Background

Huntington’s disease is an autosomal dominant inherited disorder characterized by personality changes (such as irritability and restlessness) and psychotic symptoms (such as hallucinations and delusions). When the personality changes become noticeable, involuntary movements (chorea) develop [1]. The disease is inherited through autosomal dominance and is caused by the expansion of the CAG repeat sequence in the *HTT* gene coding region. The diagnosis is based on the presence of this extension. The average age of onset of Huntington’s disease is 35–44 years. In approximately two-thirds of affected patients, neurological symptoms develop first; however, in others, the first symptoms are psychological changes. In the early stage after diagnosis, slight changes appear in eye movements, chorea movements, as do inconspicuous involuntary movements, positioning of the head, depressive symptoms, and irritability [2, 3]. Chorea movements can be partially suppressed by haloperidol and tetrabenazine, the monoamine-depleting agents [1]. However, to the best of our knowledge, no drug can completely treat Huntington’s disease yet.

Herein, we report the case of a woman with Huntington’s disease in whom the symptoms progressed slowly for 7 years after the onset of old age and in whom changes over time, such as those reflected in *N*-isopropyl-*p*-iodoamphetamine–single-photon emission computed tomography (IMP-SPECT), were recorded. Also, in this case, we show that memantine had an effect on chorea, swallowing symptoms, and oral symptoms and that treatment with memantine prevented the chorea movement and the progression of this disease.

## Case presentation

**Fig. 1:**
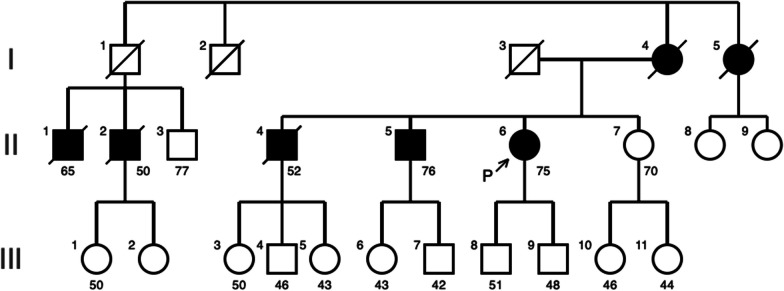
The patient’s family tree

The patient was a 75-year-old Japanese woman (II-6) who was suspected to have Huntington’s disease (because she exhibited chorea in her neck and upper limbs and had a family history of the disease) on her first visit to our hospital 2 years earlier (at age 73 years). Genetic testing was performed (with the patient’s consent) and revealed a repeat abnormality (42 repeat) of the *HTT* gene; thus, she was diagnosed with Huntington’s disease. Psychological test results were as follows: 25 points on the Mini-Mental State Examination and 10 points on the Alzheimer’s disease Assessment Scale—Cognitive Subscale. Tetrabenazine (the only drug capable of preventing disease progression) was then administered; however, nausea and fatigue necessitated the discontinuation of oral administration. The patient’s older brother (II-5) who developed the disease at the same age as she did had rapidly-progressing chorea; 5 years later, he was almost immobile and bedridden (Fig. 1). She took memantine for 5 years but did not experience any special adverse effects.

**Fig. 2:**
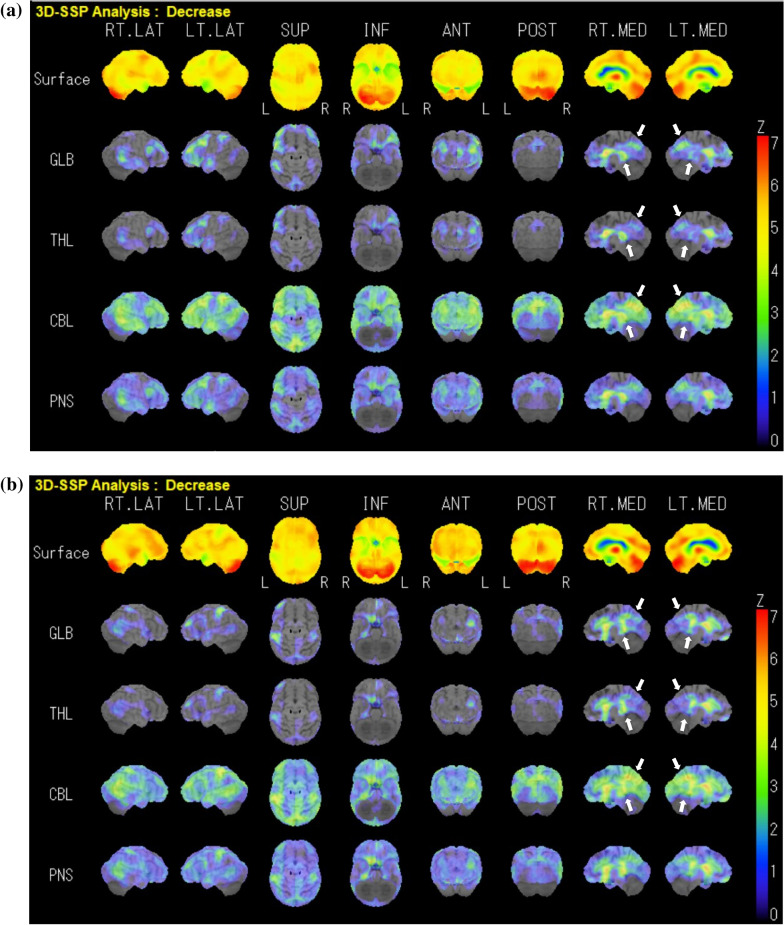
**a** *N*-isopropyl-*p*-iodoamphetamine–single-photon emission computed tomography three-dimensional stereotactic surface projection showing a decrease in cerebral blood flow on the date of diagnosis (at the age of 75 years). The white arrows indicate the posterior cingulate gyrus and precuneus, where blood flow is reduced in Alzheimer's disease. **b** Follow-up data (at the age of 80 years). The blood flow of white arrows is decreased

Magnetic resonance imaging of the head revealed changes in the striatum. In a cerebral blood flow test (IMP-SPECT), decreased blood flow was observed in the precuneus (anterior wedge) and posterior cingulate gyrus (Fig. 2). Usually, such areas of decreased blood flow are observed in patients with Alzheimer’s-type dementia. Therefore, we started treatment with an anti-dementia drug as would be done for a case of Huntington’s disease combined with Alzheimer’s disease.

Initially, donepezil administration was considered; however, because the patient was irritable, a regimen of low-dose memantine (5 mg/day) was started. Thereafter, the patient’s chorea, swallowing symptoms, and oral symptoms improved. A total of 3 months later, the dose of memantine was increased to 10 mg/day; however, the chorea worsened, so the dose was reduced to 5 mg/day. A total of 5 years since then, clinical manifestations have not worsened. The chorea movements also have not worsened substantially, and we have recorded the patient’s gait videos at the age of 75 years (Additional file 1: Video S1a) and 80 years (Additional file 2: Video S1b). The patient did not show progression in gait speed and balance. Also, after 5 years, IMP-SPECT revealed improved blood flow images in the frontotemporal lobe. Additionally, the information provided by family members and medical examinations by physicians specializing in rehabilitation did not reveal any deterioration in the patient’s swallowing function or decrease in her activities of daily living.

## Discussion and conclusions

The patient in this case demonstrated improvement in the symptoms of Huntington’s disease after the administration of oral memantine according to video recordings and cerebral blood flow tests over 7 years. The cognitive and behavioral symptoms exhibited by patients with Huntington’s disease are more similar to those exhibited by patients with frontotemporal dementia than those exhibited by patients with Alzheimer’s disease [4, 5]. Therefore, in our patients, cerebral blood flow examination indicated Alzheimer’s-type dementia. In general, many patients with Huntington’s disease show cognitive decline. Over time, involuntary movements increase, and the patient’s communication ability is affected by the deterioration of voluntary cognitive writing ability and speech. Thus, although the patient may understand the content of the evaluator’s questions, involuntary movements causing dysarthria may impair expression and communication [5]. Therefore, we believe that, in patients with Huntington’s disease who are at an old, advanced age, IMP-SPECT should be performed. Because elderly people may exhibit different types of dementia (such as Alzheimer’s-type dementia, Lewy body dementia, and frontotemporal dementia). IMP-SPECT revealed that our elderly female patient with Huntington’s disease also had Alzheimer’s-type dementia.

Recently in Japan, tetrabenazine reportedly improved Huntington’s-disease-associated chorea; however, clinical manifestations other than chorea did not improve [6]. Tetrabenazine depletes monoamines (dopamine, serotonin, and noradrenaline) at nerve endings by selectively inhibiting monoamine vesicle transporter 2 at presynaptic receptors of the central nervous system [7]. The effect of tetrabenazine on chorea results mainly from dopamine depletion in the striatum, which is the main lesion site of Huntington’s disease. However, in our patient, the oral administration of this drug caused nausea and fatigue, and the drug had to be discontinued as a result. So, considering her dementia symptoms and IMP-SPCT results, we prescribed 5 mg of memantine because it has demonstrated its efficacy in Alzheimer’s-type dementia.

Memantine is an *N*-methyl-d-aspartate (NMDA) receptor inhibitor that is usually administered to improve cognitive function in Alzheimer’s-type dementia [8]. Recently, its additional effects such as the improvement of swallowing function, lip dyskinesia, and chorea-like symptoms have been reported [8]. Several patients with Huntington’s disease were administered 20 mg of memantine and demonstrated improvement thereafter [9]. Moreover, two memantine clinical trials are ongoing for Huntington’s disease [10, 11]. This drug has also been used in treating other neurodegenerative diseases. The NMDA receptor antagonist, amantadine, has long been used not only as an anti-parkinsonism drug but also in the treatment of dyskinesia [12].

Tetrabenazines, which are involved in the regulation of monoamine receptors, have recently been approved by the US Food and Drug Administration for treating tardive dyskinesia [13, 14] and implicate a possible mechanism for chorea in Huntington’s disease. Similarly, the administration of memantine, an NMDA receptor inhibitor, may alleviate motor symptoms because monoamines and NMDA receptor regulation are closely related in the brain.

In our patient, dysphagia and dysarthria were not exacerbated for 7 years after the oral administration of 5 mg of memantine. Most memantine clinical trials focusing on Huntington’s disease have been evaluations of cognitive improvement. To the best of our knowledge, no detailed observational study of motor symptoms has been conducted [10]. The patient’s older brother (II-5 in Fig. 1) developed the disease at about the same time as she did; however, it progressed more rapidly in her brother (over 5 years). He frequently had aspiration pneumonia and was unable to walk. Thereafter, he died of this pneumonia the third time it occurred, which was 5 years after the onset of the disease. Conversely, our patient (II-8 in Fig. 1) remained ambulatory and could maintain activities of daily living, even to the extent of taking a bath and using the toilet without assistance.

Memantine has been reported to induce clinical manifestations such as myoclonus [15]; conversely, it is effective against other motor symptoms and dystonia [16]. One of the sites targeted by memantine is thought to be the dysfunctional glutamatergic neurotransmitter system via the NMDA receptor [17, 18]. In our patient, a small amount of memantine may have been effective in slowing down the progression of Huntington’s disease.

Memantine was reportedly effective in a small number of patients with Huntington’s disease [9]; in patients with the *HTT* gene who are yet to develop symptoms, memantine may suppress the future worsening of symptoms [11]. Findings in our patient suggest that oral memantine has some influence on the motor symptoms of Huntington’s disease.

We recorded changes in the results of the IMP-SPECT and videos of the patient’s gait over 5 years. To the best of our knowledge, this is the first report stating that memantine has an ameliorating effect on chorea movements in Huntington’s disease. We believe this record will provide clinicians with valuable information regarding the diagnosis and treatment of this disease.

### Supplementary Information

**Additional file 1: Video S1. (a)** Chorea symptom video on the date of diagnosis (at the age of 75 years).

**Additional file 2: Video S1. (b)** Chorea symptom video at the follow-up date (at the age of 80 years).

## Data Availability

Upon written request, the corresponding author will provide the data used to support the findings and conclusions.

## Abbreviations

IMP-SPECT: *N*-isopropyl-*p*-iodoamphetamine–single-photon emission computed tomography
NMDA: *N*-methyl-d-aspartate
